# Clinical Characteristics and Prognosis of Patients with Multi-Vessel Coronary Spasm in Comparison with Those in Patients with Single-Vessel Coronary Spasm

**DOI:** 10.3390/jcdd9070204

**Published:** 2022-06-28

**Authors:** Hiroki Teragawa, Chikage Oshita, Yuko Uchimura

**Affiliations:** Department of Cardiovascular Medicine, JR Hiroshima Hospital, 3-1-36, Futabanosato, Higashi-ku, Hiroshima 732-0057, Japan; chikage-ooshita@jrhh.or.jp (C.O.); yuuko-uchimura@jrhh.or.jp (Y.U.)

**Keywords:** cystatin-C, coronary spasm, coronary spastic angina, fasting blood sugar, multi-vessel spasm, single-vessel spasm, vasospastic angina

## Abstract

(1) Background: We have sometimes experienced patients with vasospastic angina (VSA) who presented multi-vessel spasm (MVS) on coronary angiography and spasm provocation test (SPT). However, the clinical characteristics of VSA patients with MVS and the prognosis of such patients in the clinical setting have not been clarified. Therefore, we compared the clinical characteristics and prognosis in VSA patients with MVS with those in VSA patients with single-vessel spasm (SVS). (2) Methods: A total of 152 patients (mean age, 67 years, 74 men and 78 women) with VSA, in which the presence of coronary spasm was assessed in both left coronary artery (LCA) and right coronary artery (RCA) on SPT, were enrolled. We defined VSA as the presence of >90% narrowing of the epicardial coronary artery on angiograms, accompanied by usual chest symptoms and/or ischaemic ST-T changes on the electrocardiogram. On SPT, MVS was defined as the presence of spasms on ≥2 major coronary arteries. Based on the presence of MVS, patients were divided into the MVS group and the SVS group. The frequencies of conventional coronary risk factors, blood chemical parameters, average times of anginal attack, SPT findings such as spasm provocation induced by a low dose of acetylcholine (L-ACh) and total occlusion due to coronary spasm (TOC), number of coronary vasodilators at discharge and major cardiovascular events (MACE, including cardiac death and readmission due to any cause of cardiovascular diseases) were compared between the two groups. (3) Results: The MVS and SVS groups were comprised of 98 (64%) and 54 (36%) patients, respectively. The level of fasting blood glucose (FBS) was lower (*p* < 0.01), and the level of cystatin-C (*n* = 89) tended to be higher (*p* = 0.07) in the MVS group than in the SVS group. The frequencies of L-ACh-induced coronary spasm (33% in MVS and 17% in SVS, *p* = 0.04) and TOC (12% in MVS, 0% in SVS, *p* < 0.01) were higher in the MVS group than in the SVS group. The average number of coronary vasodilators at discharge was higher in the MVS group (1.2 ± 0.4) than in the SVS group (0.9 ± 0.5, *p* < 0.01). The frequency of MACE was not different between the two groups. (4) Conclusions: Patients with MVS may have higher VSA activity on SPT and have more aggressive medications, leading to a comparable prognosis in VSA patients with SVS. MVS is an important indicator of at least VSA activity, and cardiologists should confirm this in SPT whenever possible. Further studies should confirm whether lower FBS levels and higher cystatin-C levels are any markers of MVS.

## 1. Introduction

Vasospastic angina (VSA) is characterised by the transient vasoconstriction of the epicardial coronary arteries, leading to myocardial ischaemia [1,2]. With the modification of risk factors such as smoking and intake of coronary vasodilators such as calcium-channel blockers (CCB), the prognosis of VSA has been considered relatively good in general [3]. However, in the clinical setting, medically refractory VSA or VSA with a poor prognosis was reported [3,4]. Moreover, some of the risk factors for prognosis in VSA include the non-intake of CCB, presence of coronary artery disease, ST elevation on electrocardiogram (ECG) or variant angina, history of out-hospital cardiac arrest, smoking, angina at rest, multi-vessel spasm (MVS), use of beta-blockers, focal spasm and coronary spasm in the left anterior descending coronary artery (LAD) [5,6,7,8].

Regarding MVS, we have experienced some patients demonstrating a relationship between MVS and poor prognosis or fatal cardiac events [9,10]. Although several papers have investigated the clinical characteristics in patients with MVS [11,12,13,14], such information may not be fully clarified.

Thus, to confirm the clinical characteristics and prognosis in patients with MVS, we retrospectively investigated those in patients with MVS and compared those in patients with single-vessel spasm (SVS) at our institution.

## 2. Materials and Methods

### 2.1. Study Population

**Figure 1:**
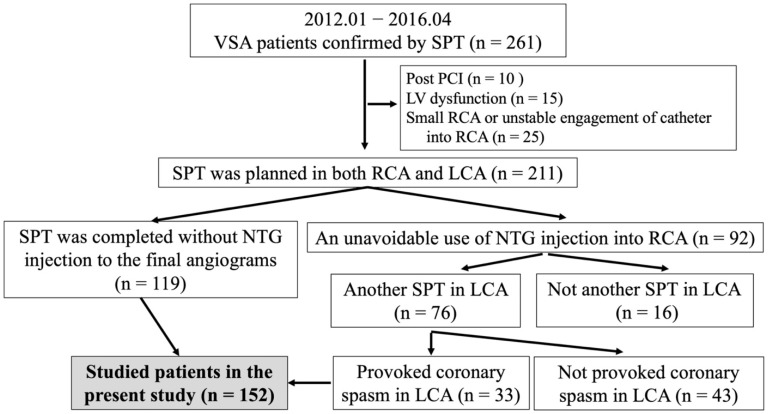
This is a figure of the study flowchart. LCA, left coronary artery; LV, left ventricle; NTG, nitroglycerin; PCI, percutaneous coronary intervention; RCA, right coronary artery; SPT, spasm provocation test; VSA, vasospastic angina.

This study was an observational and retrospective study. At our institution, from Jan 2012 to April 2016, of 1395 patients who underwent coronary angiography (CAG), 382 patients (27%) underwent a spasm provocation test (SPT) to investigate chest pain at rest or on exertion, or both. Of these, 261 patients (68%) were diagnosed with VSA due to positive SPT. We excluded patients with a previous history of percutaneous coronary intervention (*n* = 10) or left ventricular wall motion abnormality (*n* = 15). During the study period, spasm provocation at our institution was performed first in the right coronary artery (RCA). To assess the presence of MVS among the three coronary vessels, we also excluded patients for whom spasm provocation could not be performed in the RCA because of its small size or the inability to place a catheter into the ostium of the RCA (*n* = 25). Of the 211 patients whose SPT was planted in both RCA and left coronary artery (LCA), an unavoidable use of nitroglycerin (NTG) with 0.3 mg was administered intracoronary for prolonged coronary spasm or unstable haemodynamics due to coronary spasm (*n* = 92, 43%). Among 92 patients with an unavoidable use of NTG, there were 16 patients who underwent SPT only in the RCA because of a positive SPT. Among the 76 patients with another SPT for LCA, 33 had positive SPT in the LCA. Finally, 152 patients, including 119 patients who had completed SPT without NTG for the final angiograms and the aforementioned 33 patients who had positive SPT in the LCA despite unavoidable use of NTG in the RCA, were enrolled in the present study (Figure 1). The study protocol was approved by the ethics committee of our institution. Written informed consent was obtained from all the patients.

### 2.2. SPT

We conducted an SPT utilising the procedures previously published [15]. Briefly, 20 and 50 µg of acetylcholine (ACh) were injected into the RCA following the initial CAG. A maximal dose of 80 µg of ACh was injected into the RCA when coronary spasm was not elicited by 50 µg of ACh. Each time, chest symptoms and electrocardiographic changes were evaluated. CAG was acquired shortly after the induction of coronary spasms or the completion of the maximum ACh infusion. An SPT of the LCA was performed without intracoronary injection of NTG into the RCA if a coronary spasm was elicited but resolved spontaneously. In these circumstances, CAG was repeated once the SPT for the LCA was completed and NTG was injected into the RCA. Intracoronary injection of 0.3 mg of NTG was employed to reduce coronary spasms induced by ACh infusion into the RCA if they were persistent or severe enough to cause haemodynamic instability. The LCA was then subjected to an SPT. Moreover, 50 and 100 µg ACh were infused into the LCA to perform the SPT. A maximum of 200 µg of ACh was injected into the LCA if coronary spasm was not induced by 100 µg of ACh. CAG was conducted shortly after a coronary spasm had been induced or after the maximum ACh infusion had been completed. After the last CAG for the LCA, intracoronary infusion of 0.3 mg of NTG was made. Small dosages of catecholamines were administered intracoronary or intravenously if the patient’s haemodynamics remained unstable. Low doses of ACh (L-ACh) were used in this study, with the RCA and LCA receiving 20 µg and 50 µg, respectively [15].

### 2.3. Definition of Spasm-Related Factors

The coronary artery diameters were measured as previously described [16]. Atherosclerotic lesions were identified as those with stenosis of more than 20%. We checked a myocardial bridge, which was defined as a systolic constriction of the coronary artery diameter by >20% compared with that in diastole because a link between myocardial bridges and VSA has been reported [17,18]. Angina with a spontaneous ST elevation on the ECG was characterised as variant angina (VA). During the SPT, VSA was defined as a 90% constriction of the coronary arteries on angiograms, as well as the presence of typical chest discomfort and/or ST-segment deviation on ECG [3]. MVS was defined as coronary spasms that occurred in ≥2 major coronary arteries, and SVS was defined as coronary spasms that occurred in only one coronary artery. We were unable to determine when the subsequent SPT was negative after an unavoidable use of NTG in patients with MVS; hence, these individuals were excluded from the study. A focal spasm was defined as a transient vessel narrowing of >90% within the borders of one isolated coronary segment as defined by the American Heart Association. A diffuse spasm was defined as a 90% diffuse vasoconstriction observed in ≥2 adjacent coronary segments of the coronary arteries [19]. In the case of MVS, a focal spasm that occurred in either coronary artery was evaluated as having a focal spasm. We also checked the following parameters: frequencies of coronary spasms that occurred in the RCA, left anterior descending coronary arteries (LAD) and left circumflex coronary arteries (LCX); frequencies of L-ACh-induced spasms, total occlusion (TOC) of a coronary artery due to spasm; ST elevation on ECG and severe complications including prolonged unstable haemodynamics requiring intravenous catecholamines, ventricular fibrillation and pulseless ventricular tachycardia.

### 2.4. Parameters

Regarding the patients’ characteristics, information regarding the patient’s current smoking status and any family history of coronary artery disease (FH-CAD) was collected. Hypertension was defined as a systolic blood pressure of ≥140 mmHg, a diastolic blood pressure of ≥90 mmHg, or the use of antihypertensive medication. Blood chemical parameters were checked in the morning fasting state before CAG. We measured the levels of triglycerides, low-density lipoprotein cholesterol, fasting blood sugar (FBS), haemoglobin A1c (HgA1c), creatinine, C-reactive protein (CRP), brain natriuretic peptide (BNP) and cystatin-C. We also measured the D-dimer level with a lower cut-off level of 0.5 μg/mL, and a value <0.5 μg/mL was counted as 0.5 μg/mL. The estimated glomerular filtration rate (eGFR, mL/min/1.73 m^2^) was calculated using the standard formula, and the presence of CKD was defined using standard criteria [20]. Dyslipidaemia was defined as a low-density lipoprotein cholesterol level of ≥120 mg/dL or the use of medications for dyslipidaemia. Diabetes mellitus (DM) was defined as an FBS sugar level of ≥126 mg/dL, Hg A1c level of ≥6.5%, or use of anti-DM medications. Cardiac ultrasonography was used to determine the left ventricular ejection fraction (LVEF). As previously reported [21], flow-mediated dilation (FMD) as an endothelium-dependent function and NTG-induced dilation (NID) as an endothelium-independent function were assessed.

Regarding the VSA-related parameters, anginal attacks that occurred at rest or on exertion, number of anginal attacks per month, and other serious symptoms (such as cold sweating and syncope) or frequency of VA were checked. In some cases, only one anginal attack occurred, but in these cases, the number of anginal attacks was calculated as the number of anginal attacks per month estimated from the time of chest pain onset to hospitalisation.

Patients were followed up at our institution as much as feasible after discharge, and all study patients were seen for at least one follow-up. The final data collection period was set to run through March 2022. The follow-up assessments included information about patients’ medications from their medication diaries who had a recent follow-up. In the previous 3 months, we counted the number of coronary vasodilators used and the number of angina attacks (per month). The number of coronary vasodilators was evaluated at admission, discharge, and final follow-up. In each patient, cardiac events were documented, including readmission for angina or other cardiovascular disorders, and death from cardiac causes or readmission for cardiovascular reasons were considered major adverse cardiac events (MACEs).

Since this paper contains many abbreviations, a table of abbreviations is included in Supplementary Materials Table S1.

### 2.5. Statistical Analyses

Non-normally distributed data and noncontinuous variables are reported as mean with standard deviation (SD) or medians with interquartile ranges, respectively. Student’s unpaired *t*-tests, Wilcoxon signed-rank tests, or these two analyses were used to compare groups’ baseline characteristics. The Kaplan–Meier survival curve was used, along with the log-rank test, to determine MACEs. These analyses were also applied to the analysis according to the number of spastic vessels or to the analysis of patients who completed the SPT without NTG injection to the final angiograms (*n* = 119). A significant *p* value was set at 0.05.

## 3. Results

### 3.1. Patients’ Characteristics and VSA-Related Symptoms

The study included 98 patients with MVS (64%) and 54 patients with SVS (36%). No significant differences were found in the conventional coronary risk factors in the two groups (Table 1). The frequency of DM was not different between the two groups (*p* = 0.13). The frequencies of statin and antiplatelet therapies were not different between the two groups. Regarding the blood chemical parameters, the level of fasting blood glucose (FBS) was significantly lower in the MVS group than in the SVS group (*p* < 0.01), whereas the level of HgA1c was comparable between the two groups (*p* = 0.45). The levels of D-dimer, CRP, and BNP were not different between the two groups. The cystatin-C level tended to be higher in the MVS group (*n* = 60) than in the SVS group (*n* = 29, *p* = 0.07). Regarding echographic parameters, LVEF, FMD, and NID were not different between the two groups (Table 2).

Regarding VSA-related symptoms, no significant differences were noted between the two groups in the number of anginal attacks per month and other serious symptoms, such as cold sweating and syncope, or frequency of VA, whereas anginal attacks that occurred at rest, on exertion, and both at rest and exertion tended to be significantly different (*p* = 0.08). The number of coronary vasodilators at admission tended to be lower in the MVS group than in the SVS group (*p* = 0.09) (Table 3).

### 3.2. CAG and SPT Findings

The frequencies of the presence of atherosclerotic lesions and myocardial bridge were not different between the two groups (Table 4). Moreover, the presence of focal spasms was not different between the two groups. The distribution of coronary spasms in the three coronary arteries was quite different, and the frequency of coronary spasms in each coronary artery was significantly higher in the MVS group than in the SVS group. The frequencies of L-ACh-induced coronary spasm (*p* = 0.04), TOC caused by coronary spasm (*p* < 0.01) and ST elevation on ECG (*p* = 0.03) were significantly higher in the MVS group than in the SVS group. In the MVS group, 81 patients had two-vessel spasms and 13 patients had three-vessel spasms. The MVS group included 33 patients who had positive SPT findings in the LCA after an unavoidable use of NTG for RCA spasms. The frequency of severe complications was comparable between the two groups. The number of coronary vasodilators at discharge was significantly higher in the MVS group than in the SVS group (*p* < 0.01, Table 3). In the MVS group, 1 patient (1%) took no coronary vasodilators, 82 patients (84%) took 1 coronary vasodilators, 14 patients (14%) took 2 coronary vasodilators, and 1 patient (1%) took 3 coronary vasodilators. On the other hand, in the SVS group, 8 patients (15%) took no coronary vasodilators, 44 patients (81%) took one coronary vasodilator, 1 patient (2%) took two coronary vasodilators, and 1 patient (2%) took coronary vasodilators (*p* < 0.01).

### 3.3. Prognosis

**Figure 2:**
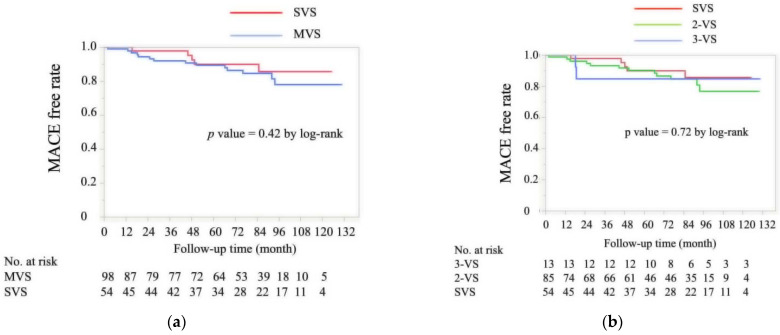
Kaplan–Meier curve for MACE-free survival during the follow-up period for the MVS and SVS groups (**a**) and for the 3-VS, 2-VS and SVS groups (**b**). 2-VS, two-vessel spasm; 3-VS, three-vessel spasm; MACE, major adverse cardiac event; MVS, multi-vessel spasm: SVS, single-vessel spasm.

The median follow-up period was 82 (52, 99) months, and the occurrence of MACE was not different between the two groups (Figure 2a). The average number of anginal attacks per month and numbers of coronary vasodilators at the final follow-up were not different between the two groups (*n* = 78 and 42 in the MVS and SVS groups, respectively). According to the number of spasm vessels, the frequency of MACE was not different among the three groups (SVS, two-vessel spasms, and three-vessel spasms, Figure 2b). In 119 study patients who completed SPT without intracoronary injection of NTG to the final angiograms, the frequency of MACE was not different between the two groups (data were not shown, log-rank *p* = 0.83).

## 4. Discussion

In this study, we investigated the clinical parameters in patients with MVS, comparing those in patients with SVS. Our main findings were as follows: (1) The FBS level was lower in the MVS group than in the SVS group, and the cystatin-C level tended to be higher in the MVS group than in the SVS group, whereas other factors, such as smoking, FH-CAD, and endothelial function, were not different between the two groups. (2) VSA activity during the SPT, assessed by the presence of LAD spasm, L-ACh-induced coronary spasm, TOC, and ST elevation were higher in the MVS group than in the SVS group, leading to a higher number of coronary vasodilators at discharge. (3) During the follow-up with a median of 84 months, the occurrence of MACEs between patients with MVS and SVS was not different.

Several studies have revealed the prognostic effect of MVS on patients with VSA [6,8]. As an explanation for the relationship between MVS and poor prognosis, MVS may cause severe and extended myocardial ischaemia assessed by myocardial perfusion imaging [22], impairment of fibrinolytic activity [13], left ventricular dysfunction [23], and malignant arrhythmia [11]. In this study, we excluded patients with LV dysfunction, which may be caused by MVS, and the levels of BNP and LVEF at admission, which can be examined in routine medical care, were not different between the two groups. Our findings may not account for MVS-induced LV dysfunction. However, the higher frequencies of L-ACh-induced coronary spasm, TOC, and ST elevation on ECG may indicate the higher spasmogenic activity in each coronary vessel in the MVS group. Thus, hyperventilation is likely to induce coronary spasm in patients with MVS [11,24]; however, the hyperventilation provocation test was not routinely performed in our institution. Furthermore, exercise-induced coronary spasm was more frequent in patients with MVS [12], and this study also demonstrated that a higher frequency of chest symptoms is highly likely during exercise. In patients with MVS, depressed cardiac vagal control and sympathetic-dominant interaction were present [25], and such a mechanism may account for exercise-induced coronary spasms.

In this study, regarding blood chemical parameters, the FBS level was lower, whereas the level of cystatin-C tended to be higher. In the former, the frequency of DM, drugs taken that may affect the glucose level, such as statins, β-blockers, and diuretic drugs [26,27], were not different between the two groups (data were not shown). However, these factors may have had a combined effect. In any case, the relationship between blood glucose levels and MVS should be examined in the future. In the latter, Lee et al. [14] reported that the level of cystatin-C, which is a sensitive marker for renal function, is higher in the MVS group than in the SVS group. Some interest has focused on the cystatin-C level in VSA [28]. The relationship between a higher cystatin-C level and MVS may demonstrate the potential development of MVS-induced renal dysfunction. In some diseases, the relationship between the cystatin-C level and endothelial dysfunction [29] was reported. Regarding VSA, because endothelial dysfunction may be involved in the mechanism of VSA [21], cystatin-C may be a marker associated with coronary spasm, especially in MVS; however, whether this is a result or a cause remains to be seen. Further study is needed on this issue as well.

In this study, the prognosis was not different between patients with MVS and SVS. Higher VSA activities of MVS with higher frequencies of LAD spasm, L-ACh-induced coronary spasm, TOC, and ST elevation were assessed during the SPT, and the growing recognition that MVS is a factor influencing the prognosis of VSA [6] may lead to the aggressive medical therapy at discharge in patients with MVS. On the contrary, the number of coronary vasodilators was not different at follow-up. The VSA activity does not necessarily continue in the same manner during the disease course, and it is common to experience high anginal attacks with higher frequency at certain times of the year and low frequency at others. Even in patients with MVS, an abnormal heart rate variability decrease within 1 month [25]. In addition, we checked only the number of coronary vasodilators in this study, but not the kinds, doses, and presence of generic forms of coronary vasodilators, which may affect the prognosis or occurrence of anginal attack in patients with VSA [30,31]. Furthermore, lifestyle modifications for smoking or emotional stress were not also checked; these were important prognostic factors for coronary spasm [32,33]. These factors may affect the prognosis of patients with MVS and SVS.

This study has an important clinical implication. SPT is an important examination; although it does not diagnose VSA, it can determine the VSA activity or provide prognostic information on several factors, such as the presence of significant coronary stenosis, LAD spasm, focal spasm, and MVS [5,6,7,8]. In this study, the prognosis of MVS was not different when compared from that of SVS; however, the VSA activity in MVS, undoubtedly, was higher than that in SVS. Thus, to clarify the presence of MVS, SPT should be performed on both LCA and RCA, even in cases with an unavoidable use of NTG as much as possible. Sueda et al. [34] reported that SPT should start from the provocation into the LCA to assess MVS. This is an important message, and we have started SPT from the LCA these days.

This study has several limitations. First, this study had a small sample size and was retrospectively performed at only one institution. The characteristics of the patients with MVS may not account for all the patients with VSA, and these will be confirmed in further studies. Second, patients who had no coronary provocation in the LCA after an unavoidable use of NTG for the RCA spasm were excluded, and these patients may account for the patients with MVS. Thus, based on such results, the frequency of MVS and patients’ characteristics may be different. Third, as mentioned above, patients whose SPT was started from the RCA were included in this study because of their long-term follow-up. Forth, Seattle Angina Questionnaire (SAQ) and other methods are available to evaluate the quality of chest pain, but we were not able to evaluate them in this study. Finally, we failed to follow the patients closely enough, and we may not have collected sufficient prognostic information.

## 5. Conclusions

We investigated the clinical parameters in patients with MVS in comparison with those in patients with SVS. The VSA activity during the SPT was higher in the MVS group than in the SVS group, leading to the aggressive medication and a similar prognosis in such patients. Thus, cardiologists should consider the VSA activity and possible prognostic factors of MVS and should try to detect the presence of MVS in SPT.

## Figures and Tables

**Table 1:** Patients’ characteristics.

	SVS	MVS	*p* Value
Number (%)	54 (36)	98 (64)	
Male/Female	23/31	51/47	0.26
Age (years)	67 ± 11	68 ± 11	0.63
Body mass index	23.9 ± 3.3	24.7 ± 4.3	0.24
Smoking (%)	9 (17)	21 (21)	0.32
Hypertension (%)	42 (78)	66 (67)	0.17
Dsylipidemia (%)	36 (66)	61 (62)	0.58
Diabetes mellitus (%)	17 (31)	20 (20)	0.13
FH-CAD (%)	8 (15)	26 (27)	0.10
CKD (%)	16 (30)	31 (32)	0.80
Taking statins (%)	25 (46)	38 (39)	0.37
Taking antiplatelet therapy (%)	10 (19)	28 (29)	0.17

Numbers were expressed as the numbers (percentage) and values were expressed as the mean with standard deviation. CKD, chronic kidney disease; FH-CAD, family history of coronary artery disease; MVS, multi-vessel spasm; SVS, single-vessel spasm.

**Table 2:** Blood chemical and echographic parameters.

	SVS	MVS	*p* Value
Blood chemical parameters			
Total cholesterol (mg/dL)	200 ± 29	194 ± 34	0.28
Triglyceride (mg/dL)	136 ± 56	137 ± 76	0.70
HDC-cholesterol (mg/dL)	59 ± 15	59 ± 17	0.76
LDL-cholesterol (mg/dL)	115 ± 29	108 ± 27	0.17
Fasting blood sugar (mg/dL)	109 ± 20	101 ± 16	<0.01
Hemoglobin A1c (%)	6.0 ± 0.8	5.9 ± 0.8	0.45
D-dimer (μg/mL)	0.5 (0.5, 0.9)	0.6 (0.5, 1.1)	0.58
C-reactive protein (mg/dL)	0.06 (0.02, 0.13)	0.06 (0.03, 0.25)	0.33
eGFR (mL/min/1.73 m^2^)	71.9 ± 15.5	70.0 ± 15.5	0.48
Brain natriuretic peptide (pg/mL)	20 (14, 43)	21 (11, 45)	0.85
Cystatin C (mg/L)	0.92 ± 0.14 (*n* = 29)	0.99 ± 0.21 (*n* = 60)	0.07
Echographic parameters			
LVEF on echocardiograpy (%)	65 ± 8	66 ± 10	0.35
FMD on brachial ultrasonography (%)	4.2 ± 3.2	3.4 ± 3.2	0.17
NID on brachial ultrasonography (%)	14.0 ± 5.9	15.0 ± 7.1	0.57

Numbers were expressed as the numbers (percentage) and values were expressed as the mean with standard deviation or the median with interquartile ranges. eGFR, estimated glomerular filtration ratio; FMD, flow-mediated dilation; HDL, high-density lipoprotein; LDL, low-density lipoprotein; LVEF, left ventricular ejection fraction, MVS, multi-vessel spasm; NID, nitroglycerin-induced dilation; SVS, single-vessel spasm.

**Table 3:** VSA-related parameters.

	SVS	MVS	*p* Value
Chest symptom			0.08
At rest (%)	48 (89)	76 (78)	
On exertions (%)	5 (9)	10 (10)	
Both at rest and exertion (%)	1 (2)	12 (12)	
Variant angina (%)	0 (0)	2 (2)	0.29
Other serious symptoms (%)	4 (7)	8 (8)	0.87
Number of chest symptoms per month			
On admission	4 (2, 11)	4 (2, 10)	0.50
Follow-up	0 (0, 1) (*n* = 43)	0 (0, 3) (*n* = 78)	0.16
Number of taking vasodilator			
On admission	0.6 ± 0.6 1 (0,1)	0.5 ± 0.6 0 (0, 1)	0.09 0.05
At discharge	0.9 ± 0.5 1 (1, 1)	1.2 ± 0.4 1 (1, 1)	<0.01 <0.01
Follow-up	1.3 ± 0.9 1 (1, 2) (*n* = 43)	1.6 ± 0.9 1 (1, 2) (*n* = 78)	0.05 0.09

Numbers were expressed as the numbers (percentage) and values were expressed as the mean with standard deviation or the median with interquartile ranges. MVS, multi-vessel spasm; SVS, single-vessel spasm.

**Table 4:** The results of CAG and SPT.

	SVS	MVS	*p* Value
CAG			
Atherosclerotic lesion (%)	31 (57)	65 (66)	0.28
Myocardial bridge (%)	4 (7)	11 (11)	0.45
SPT			
2-vessel spasms and 3-vessel spasms	None	85/13	
Location			
RCA spasm (%)	6 (11)	97 (99)	<0.01
LAD spasm (%)	47 (87)	96 (98)	0.01
LCX spasm (%)	1 (2)	16 (16)	<0.01
L-ACh-induced coronary spasm (%)	9 (17)	32 (33)	0.04
TOC due to coronary spasm (%)	0 (0)	12 (12)	<0.01
Focal spasm (%)	18 (33)	27 (28)	0.45
ST elevation on electrogram (%)	3 (6)	19 (20)	0.03
Severe complications (%)	3 (6)	5 (5)	0.90

Numbers were expressed as the numbers (percentage). L-ACh, low dose of acetylcholine; LAD, left anterior descending coronary artery; LCX, left circumflex coronary artery; MVS, multi-vessel spasm; RCA, right coronary artery; SVS, single-vessel spasm; TOC, total occlusion.

## Data Availability

Not applicable.

## Supplementary Materials

The following supporting information can be downloaded at: https://www.mdpi.com/article/10.3390/jcdd9070204/s1, Table S1: Abbreviation list.
