# Supplementary material for: Clinical Characteristics and Prognosis of Patients with Multi-Vessel Coronary Spasm in Comparison with Those in Patients with Single-Vessel Coronary Spasm
Source: J Cardiovasc Dev Dis. 2022 Jun 28;9(7):204. doi: 10.3390/jcdd9070204 (PMC9322607; doi:10.3390/jcdd9070204)
Supplement: Supplementary file 1 [file jcdd-09-00204-s001.zip › jcdd-1775218-supplementary.pdf]

Table S1. Abbreviation list.

ACh	acetylcholine
BNP	brain natriuretic peptide
CAG	coronary angiography
CCB	calcium-channel blockers
CKD	chronic kidney disease
CRP	C-reactive protein
DM	diabetes mellitus
ECG	electrocardiogram
eGFR	estimated glomerular filtration rate
FBS	fasting blood sugar
FH-CAD	family history of coronary artery disease
FMD	flow-mediated dilation
HDL	high-density lipoprotein
HgA1c	Haemoglobin A1c
L-ACh	a low dose of acetylcholine
LAD	left anterior descending coronary artery
LCA	left coronary artery
LCX	left circumflex coronary artery
LDL	low-density lipoprotein
LVEF	left ventricular ejection fraction
MACE	major cardiovascular events
MVS	multi-vessel spasm
NID	nitroglycerin-induced dilation
NTG	nitroglycerin
RCA	right coronary artery
SD	standard deviation
SPT	spasm provocation test
SVS	single-vessel spasm
TOC	total occlusion due to coronary spasm
VA	variant angina
VSA	vasospastic angina
2-VS	two-vessel spasm
3-VS	three-vessel spasm
